# A short tutorial in analyzing NGS data of cancer genomes for somatic mutation calling

**DOI:** 10.1186/1471-2105-14-S17-A15

**Published:** 2013-10-22

**Authors:** Huy Vuong, Zhongming Zhao

**Affiliations:** 1 Department of Biomedical Informatics, Vanderbilt University, Nashville, TN 37232, USA; 2 Department of Psychiatry, Vanderbilt University, Nashville, TN 37232, USA; 3 Department of Cancer Biology, Vanderbilt University, Nashville, TN 37232, USA; 4 Center for Quantitative Sciences, Vanderbilt University School of Medicine, Nashville, TN 37232, USA

## Background

Somatic mutation is the key element of tumorigenesis as these changes in nucleotide sequence of the cancer genome in somatic cells acquired throughout life can lead to protein alteration, cellular damage and thus cause cancer. The advent of next generation sequencing has significantly improved our ability to identify somatic mutations in cancer genomes paving the way for the comprehensive online catalogue for somatic mutation in human cancer (COSMIC)^1^ which contains more than 820,000 mutations so far. Nevertheless, there are still many challenges in detecting somatic mutation in cancer especially for low frequency mutation due to either tumor heterogeneity or contamination with normal cells. Here, in this short tutorial, I will present a recent somatic mutation caller tool developed by the Broad Institute called Mutect^2^ as part of the GATK (Genome Analysis Toolkit). I will use my own NGS dataset to demonstrate the tools and address some issues of troubleshooting input data and interpreting output.

## Materials and methods

1. MuTect can be downloaded here: http://www.broadinstitute.org/cancer/cga/mutect. You must register an account with Broad Institute to download.

2. Instruction for running MuTect: http://www.broadinstitute.org/cancer/cga/mutect_run

3. User support forum: http://gatkforums.broadinstitute.org/categories/mutect

4. Because this workshop uses real world NGS data set, it’s not required for participants to follow the walkthrough. However, participants are encouraged to run MuTect with their own data later (whole genome sequencing, whole exome sequencing, RNA sequencing data, etc.)
